# Aggressive prolactinomas in men are associated with visual disturbances and pituitary hormone deficiencies

**DOI:** 10.1038/s41598-025-23646-z

**Published:** 2025-11-11

**Authors:** Everardo J. Díaz-López, Rocío Villar-Taibo, Eva Fernández-Rodríguez, Laura Cotovad-Bellas, Alberto Pena-Dubra, Teresa Prado-Moraña, Gemma Rodríguez-Carnero, Kelly Vargas-Osorio, José Manuel Cameselle-Teijeiro, Ignacio Bernabéu

**Affiliations:** 1 Department of Endocrinology and Nutrition , University Clinical Hospital of Santiago de Compostela , Compostela , Spain; 2 Center for Research in Molecular Medicine and Chronic Diseases (CIMUS) , Unidad de Enfermedades Tiroideas e Metabólicas (UETeM) University of Santiago de Compostela , Compostela , Spain; 3 Department of Endocrinology and Nutrition , University Clinical Hospital of Ourense , Ourense , Spain; 4 Department of Endocrinology and Nutrition , University Clinical Hospital of Ferrol , Ferrol , Spain; 5 Department of Pathology , University Clinical Hospital of Santiago de Compostela , Compostela , Spain

**Keywords:** Prolactinomas, Pituitary neuroendocrine tumors, Lactotroph PitNET, Aggressive tumors, Aituitary adenoma, Pituitary diseases, Endocrinology

## Abstract

Prolactinomas/lactotroph pituitary neuroendocrine tumors are ten times less frequent in men than in women and their characteristics are less well known. The latest WHO classification includes them among pituitary tumors with a high risk of recurrence. This study aimed to identify clinical parameters suggesting aggressive prolactinomas. We conducted a retrospective study in three hospitals in Galicia, Spain, including 41 men with prolactinomas. The mean age at diagnosis was 46.5 ± 16.2 years. Baseline prolactin levels were a median of 800 ng/ml, with 95% being macroprolactinomas. Aggressive prolactinomas (*n* = 10) compared to non-aggressive (*n* = 31), had higher rates of visual disturbances (60% vs. 13%; *p* = 0.005) and deficiencies of thyroid-stimulating hormone (70% vs. 13%; *p* = 0.001) and adrenocorticotropic hormone (50% vs. 7%; *p* = 0.006) at diagnosis. Prolactin levels correlated with tumor maximum diameter, more stronger in aggressive cases (*r* = 0.68; *p* = 0.047). In our study, a 24% of the prolactinomas were classified as aggressive. We found that prolactinomas in males presented with significantly elevated prolactin levels that correlate strongly with tumor diameter, as well as, visual disturbances and deficiencies of thyroid-stimulating hormone and adrenocorticotropic hormone, should raise suspicion of aggressive lactotroph pituitary neuroendocrine tumors/prolactinomas.

## Introduction

Lactotroph pituitary neuroendocrine tumors (PitNETs), commonly referred to as prolactinomas are well-differentiated tumors derived from PIT1-lineage adenohypophyseal cells with lactotroph differentiation, according to the 2022 World Health Organization (WHO) classification^1^. They account for nearly 57% of all pituitary adenomas and show a pronounced sex-related imbalance, being ten times less common in men than in women. The biological and clinical behavior in male patients, however, remains less well understood^2^. While women of reproductive age typically present with microadenomas, approximately 80% of prolactinomas diagnosed in men are macroadenomas^3^. In men, these tumors are often large and invasive, frequently associated with mass effects, hypopituitarism, and lower response rates to dopamine agonists^4,5^.

Most prolactinomas/lactotroph PitNETs respond favorably to medical therapy, particularly dopamine agonists (DAs), which remain the first-line treatment and are generally effective and well tolerated^6^. However, a subset of patients exhibit resistance or refractoriness, and a minority evolve into clinically aggressive adenomas^4^. Rising prolactin levels in previously controlled patients may signal aggressiveness and, in rare cases, malignant transformation^4^. Beyond tumor growth, macroprolactinomas—and less frequently microprolactinomas—may compromise pituitary function, making comprehensive assessment of hormone deficiencies an essential part of patient management^4^.

The concept of tumor aggressiveness was already highlighted in the 2017 WHO classification, which noted that lactotroph tumors in men may constitute a subtype of aggressive pituitary adenomas regardless of histological grade^7^. According to the European Society of Endocrinology, aggressive pituitary tumors are defined as radiologically invasive lesions with unusually rapid growth or clinically significant progression despite optimal standard therapies, including dopamine agonists, surgery, and radiotherapy^8^. In practice, aggressiveness is suspected when tumors demonstrate invasive growth with persistent hormonal hypersecretion under adequate therapy, often requiring multimodal treatment and showing high recurrence risk^6^.

Aggressive pituitary adenomas overall comprise about 10% of pituitary tumors, and are clinically relevant because of their association with morbidity and mortality even in the absence of metastases^4,9^. The true prevalence of aggressive prolactinomas is uncertain due to heterogeneous definitions, scarcity of prospective studies, and publication bias, but they appear to represent a minority^6^. Risk factors for poor therapeutic response and aggressive behavior include male sex, younger age at diagnosis, radiological and histopathological invasiveness, and proliferative markers such as Ki-67 ≥ 3%, mitotic index > 2/10 high-power fields, and p53 immunopositivity^10,11^, as well as the loss of expression of p27, ATRX and p53 alterations^12,13^. In men, prolactinomas tend to follow a more aggressive course, with higher recurrence after surgery and progression despite medical or radiotherapy treatment^14,15^.

At the molecular level, resistance to DAs may be linked to reduced D2 receptor expression or downstream signaling alterations^16^, with additional biological factors also implicated^17^. Pathological evaluation therefore remains crucial to characterizing aggressiveness^6^.

Taken together, these observations underscore the clinical importance of identifying early predictors of aggressiveness in prolactinomas. The present multicenter study seeks to characterize clinical features that may facilitate the early recognition of aggressive prolactinomas/lactotroph PitNETs. To our knowledge, studies specifically comparing aggressive versus non-aggressive lactotroph tumors are scarce, and this work aims to provide novel insights into potential predictors of aggressiveness in male patients.

## Materials and methods

### Study design

This observational, cross-sectional, multicenter, retrospective study was conducted in three tertiary university medical centers in Galicia, Spain. Forty-one male patients with a diagnosis of prolactinoma/lactotroph PitNET were included. Medical records of all patients diagnosed over the past thirty years (up to 2024) were reviewed. The study protocol was approved by the Autonomous Research Ethics Committee of Santiago-Lugo (number 2024/373).

### Diagnostic criteria

The diagnosis of prolactinoma/lactotroph PitNET was established by the presence of hyperprolactinemia markedly above the upper normal limit (> 100 ng/mL) together with radiological evidence of pituitary adenoma on magnetic resonance imaging (MRI). MRI with gadolinium contrast was the standard imaging modality; computed tomography (CT) was only used when MRI was contraindicated or unavailable. Other causes of hyperprolactinemia, such as stalk compression, were excluded. Mixed secretory tumors were not included. Tumors were classified as microadenomas (< 1 cm) or macroadenomas (≥ 1 cm)^2^.

### Data collection

The following data were extracted: demographic information, presenting symptoms, biochemical profile, imaging findings, and histopathological characteristics when available. Tumor volume was estimated using the modified ellipsoidal formula (calculated as the anteroposterior diameter multiplied by the craniocaudal diameter multiplied by the transverse diameter, divided by 2) at the time of diagnosis^18^. Pituitary hormone deficiencies, previous treatments (medical, surgical, and radiotherapy) and related outcomes were documented. Clinical manifestations related to mass effect or hyperprolactinemia were also recorded.

Baseline evaluation included thyroid-stimulating hormone (TSH), free thyroxine, follicle-stimulating hormone (FSH), luteinizing hormone (LH), adrenocorticotropic hormone (ACTH), testosterone, cortisol, and insulin-like growth factor-1 (IGF-1). Hormone levels were measured in local hospital laboratories using validated methods (radioimmunoassay, immunoradiometry, or enzyme immunoassay), with reference ranges specific to each assay. Assessments were performed at diagnosis, during follow-up, and at the last clinic visit.

### Treatment evaluation

Medical treatment with DAs was analyzed in terms of drug type, cumulative dose, duration, tolerability, and resistance. Surgical management was assessed regarding the approach, number of procedures, and complications. Radiotherapy characteristics, including modality and adverse effects, were also recorded.

Resistance to DAs was characterized by less than 50% reduction in tumor size despite receiving maximum conventional doses of DAs^19^.

The hormonal response was classified as complete if prolactin levels normalized, partial if there was a greater than 50% reduction in prolactin levels without normalization, or absent if no significant change was observed.

Radiological response was considered complete if MRI revealed no detectable tumor tissue after treatment. Partial response was defined as a reduction in tumor volume of more than 30%. Stability was characterized by no change in tumor volume, a decrease of less than 30% or an increase of less than 20%. Progression was defined as tumor growth greater than 20% or the appearance of new metastases^20^.

Clinical cure was defined as the achievement and sustained maintenance of normoprolactinemia for more than one year without the need for treatment, accompanied by the absence of radiological evidence indicating the presence of a pituitary tumor.

### Histopathological, immunohistochemical and molecular analysis

The surgical specimens were fixed in neutral, phosphate-buffered, 10% formalin and included in paraffin blocks. Formalin-fixed paraffin-encubedded (FFPE) tissue sections were stained with hematoxylin-eosin. Immunohistochemical stains were also performed on 4 μm thick paraffin sections using a peroxidase – conjugated – labeled dextran polymer (Dako EnVision peroxidase/DAB; Dako, Glostrup, Denmark), with 3,3`-diaminobenzidine as the chromogen, and using a series of primary antibodies as follows: PIT-1 (clone, D7; dilution 1:200, antigen retrieval, pH 9; manufacturer, Gennova, Sevilla, Spain), PRL (PRL2644, 1:300, Ph 9, Termo-Fisher, Massachusetts, US), GH (GH-2, 1:500, pH 9, Abcam, Cambridge, UK), GATA3 (L50-823, 1:50, Ph 6, BioSystems, Barcelona, Spain), Cytokeratin’s 8/18 (CK8/18) (EP17/EP30, ready to use, pH 9, Dako), estrogen receptor (EP1/IR044IVD; ready-to-use, pH 9, Dako), p27 (SX5368, 1:50, pH 9 Dako), ATRX (AX1, 1:100, Ph 9, Dianova, Hamburg, Germany), p53 (D07, ready-to-use, pH 9, Dako) and Ki67 (MIB1, 1:200; pH 9, Dako). Analysis of somatic mutations through next generations sequencing (NGS) from paraffin-embedded tissue from one of the cases (metastatic PitNET).

The samples were classified according to the criteria of the 5th edition of the WHO classification of the endocrine and neuroendocrine tumors^1^.

### Statistical analysis

A descriptive analysis was first performed to characterize the study population. Categorical variables were expressed as absolute and relative frequencies, while continuous variables were summarized using measures of central tendency and dispersion. Normally distributed continuous variables were reported as mean ± standard deviation (SD), and non-normally distributed variables as median and interquartile range (IQR).

Group comparisons for categorical variables were conducted using the chi-squared test or Fisher’s exact test. For continuous variables, normality was assessed with the Shapiro–Wilk test and homogeneity of variance with Levene’s test. Between-group comparisons were performed using the Student’s t-test for normally distributed data or the Mann–Whitney U test for non-normally distributed data. Associations between quantitative variables were explored using Pearson’s correlation coefficient for parametric data and Spearman’s rank correlation for non-parametric data. Coefficients of determination (r²) were also calculated. Odds ratios (OR) with 95% confidence intervals (CI) were estimated for categorical associations.

Regression analyses were used to adjust for potential confounding factors. Analysis of covariance (ANCOVA) was performed with age at diagnosis as a covariate, and multivariable logistic regression models adjusted for age were applied to assess associations between clinical outcomes and exposure variables. All statistical analyses were performed using IBM SPSS Statistics, version 28. A two-tailed p-value < 0.05 was considered statistically significant.

## Results

### Clinical, endocrine and radiological characteristics

Clinical, endocrine, and radiological characteristics of male patients with prolactinomas/lactotroph PitNETs are summarized in Table 1. The mean age at diagnosis was 46.5 ± 16.2 years. Hypogonadism was the most frequent clinical manifestation (53.7%), followed by headache (31.7%). Median baseline prolactin levels reached 800 ng/mL. Gonadotropin deficiency was present in 61% of patients. Most cases (95%) corresponded to macroprolactinomas, with a median maximum tumor diameter of 15.7 mm [IQR 21 mm]. Suprasellar extension was observed in 73%, sphenoidal extension in 73.2%, cavernous sinus invasion in 63.4%, and bone invasion in 12.2%.

**Table 1:** Clinical, endocrine, and radiological characteristics of male patients with prolactinomas/lactotroph PitNETs.

	Overall cohort (*n* = 41)	Aggressive (*n* = 10)	Non-aggressive (*n* = 31)	*P* value	Age-adjusted *P* value
Demographic and follow-up data					
Age at diagnosis (years, mean ± SD)	46.5 ± 16.2	43.3 ± 17.3	47.4 ± 16.1	0.494	–
Current age (years, mean ± SD)	58.8 ± 14.8	58.7 ± 12.6	58.8 ± 15.7	0.186	0.979
Follow-up (years, median [IQR])	10.0 [14.8]	10.9 [17.5]	10.0 [10.2]	0.272	0.997
Presenting complaint at first visit					
Incidental finding (n, %)	11 (26.8)	2 (20.0)	9 (29.0)	0.301	0.019
Headaches (n, %)	13 (31.7)	5 (50.0)	8 (25.8)	0.197	0.028
Visual disturbances (n, %)	10 (24.4)	6 (60.0)	4 (12.9)	0.005	0.018
Symptoms of hypogonadism (n, %)	22 (53.7)	3 (30.0)	19 (61.3)	0.203	0.294
Galactorrhea (n, %)	2 (4.9)	0	2 (6.5)	0.507	0.010
Pituitary axis impairment at diagnosis					
Somatotroph axis (n, %)	8 (19.5)	4 (40.0)	4 (12.9)	0.082	0.204
Corticotroph axis (n, %)	7 (17.1)	5 (50.0)	2 (6.5)	0.006	0.004
Thyrotroph axis (n, %)	11 (26.8)	7 (70.0)	4 (12.9)	0.001	< 0.001
Gonadal axis (n, %)	25 (61.0)	8 (80.0)	17 (54.8)	0.265	0.272
Tumor characteristics					
Prolactin (ng/mL, median [IQR])	800 [2466]	4830 [910]	651 [1524]	0.061	0.560
Tumor volume (mm³, median [IQR])	4442.2 [10528]	10556.0 [36573]	3217.5 [9205]	0.029	0.267
Maximum diameter (mm, median [IQR])	15.7 [21.0]	36.0 [28.0]	14.0 [16.5]	0.002	0.018
Extrasellar extension (n, %)	22 (53.7)	10 (100)	12 (38.7)	0.009	< 0.001
Sphenoidal extension (n, %)	30 (73.2)	9 (90.0)	12 (38.7)	0.007	0.008
Cavernous sinus invasion (n, %)	26 (63.4)	10 (100)	16 (51.6)	0.007	0.004
Bone invasion (n, %)	5 (12.2)	4 (40.0)	1 (3.2)	< 0.001	0.001

Values are expressed as mean ± SD, number of patients (percentage, calculated on group total), or median [interquartile range, IQR].

### Primary treatment and surgical indications

Treatment modalities and outcomes regarding tumor response and biochemical control are shown in Table 2. All patients initially received medical therapy, with a mean cabergoline weekly dose of 2.5 ± 2.0 mg. Overall, 26% required surgery due to resistance to medical treatment and/or extrasellar extension of the tumor; of these, 40% underwent a transsphenoidal approach and 20% a transcranial one, and 60% subsequently received radiotherapy. Surgical indications included resistance to medical treatment (12%), extrasellar extension (12%), visual disturbances (5%), tumor apoplexy (2%), and intolerance to pharmacological therapy (3%).

**Table 2:** Treatment modalities and outcomes regarding tumor response and biochemical of male patients with prolactinomas/lactotroph PitNETs.

	Overall cohort (*n* = 41)	Aggressive (*n* = 10)	Non-aggressive (*n* = 31)	*P* value	Age-adjusted *P* value
Treatment					
Maximum weekly cabergoline dose (mg, mean ± SD)	2.5 ± 2.0	2.9 ± 2.6	2.1 ± 1.5	0.005	0.001
Surgery (n, %)	11 (26.8)	6 (60.0)	5 (16.1)	0.005	0.005
Reoperation (n, %)	3 (7.3)	3 (30.0)	0	0.002	0.010
Post-surgical radiotherapy (n, %)	4 (9.8)	4 (40.0)	2 (6.5)	0.004	0.004
Outcomes					
Partial tumor control (n, %)	15 (36.6)	5 (50.0)	10 (38.7)	0.777	0.026
Partial biochemical control (n, %)	36 (87.8)	8 (80.0)	28 (90.3)	0.063	0.350
Controlled with medical therapy (n, %)	27 (65.9)	4 (40.0)	23 (74.2)	0.078	0.261
Last prolactin (ng/mL, median [IQR])	6.8 [24.05]	31.3 [241.1]	5.5 [12.4]	0.041	0.004
Residual tumor (n, %)	34 (82.9)	8 (80.0)	26 (83.9)	0.742	0.217
Mortality (n, %)	1 (2.4)	1 (10.0)	0	0.244	0.188

Values are expressed as mean ± SD, number of patients (percentage, calculated on group total), or median [interquartile range, IQR].

### Aggressive vs. non-aggressive tumors

**Fig. 1:**
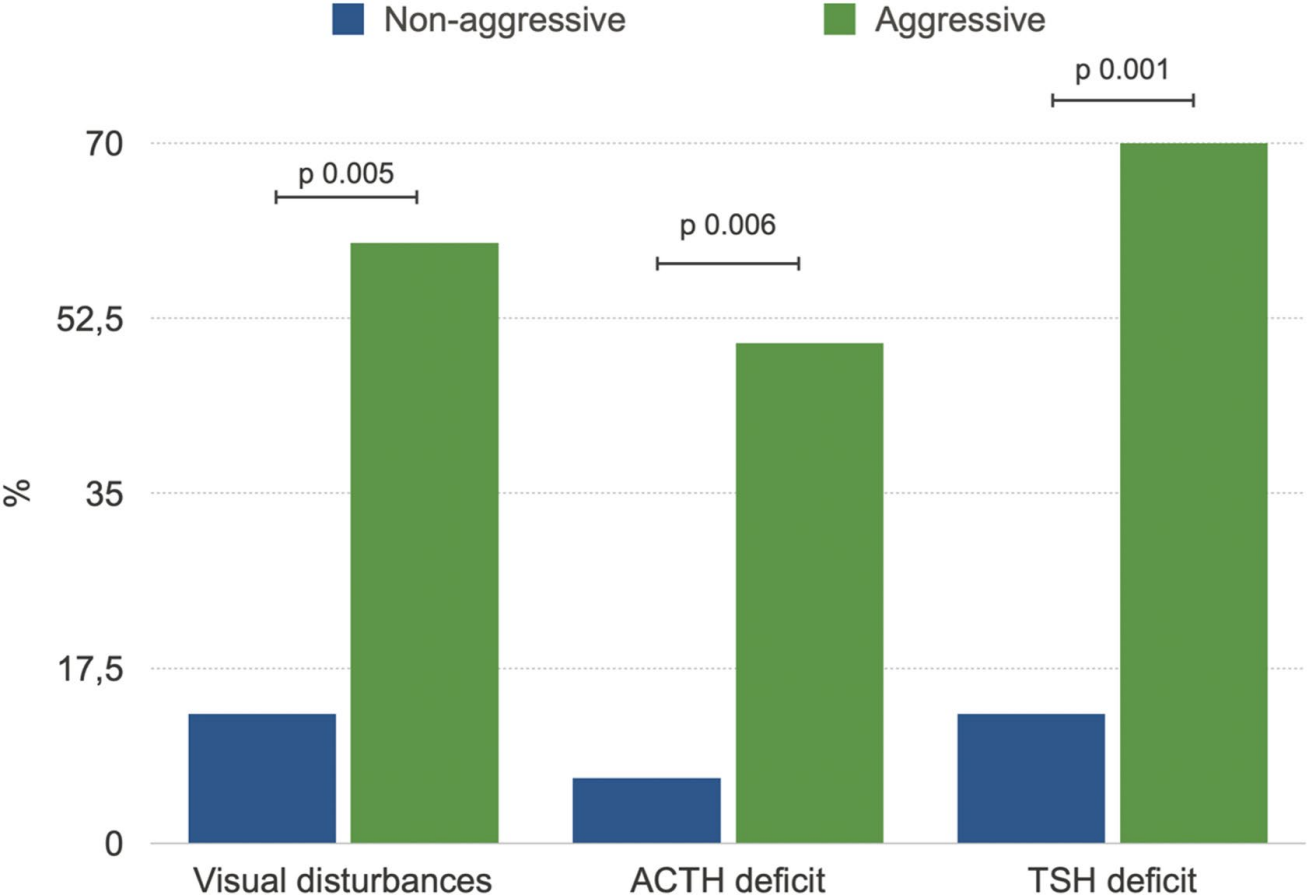
Significant differences in clinical alterations in prolactinomas/lactotroph PitNETs, persisting after adjustment for age.

In this series, aggressive prolactinomas/lactotroph PitNETs (*n* = 10), compared with non-aggressive tumors (*n* = 31), were more frequently associated with visual disturbances (60% vs. 13%; OR 13, 95% CI 2.2–74.1; *p* = 0.005), TSH deficiency (70% vs. 13%; OR 15, 95% CI 2.8–87.0; *p* = 0.001), and ACTH deficiency (50% vs. 7%; OR 14.5, 95% CI 2.1–96.0; *p* = 0.006). These associations remained statistically significant after adjustment for age at diagnosis (Fig. 1).

**Fig. 2:**
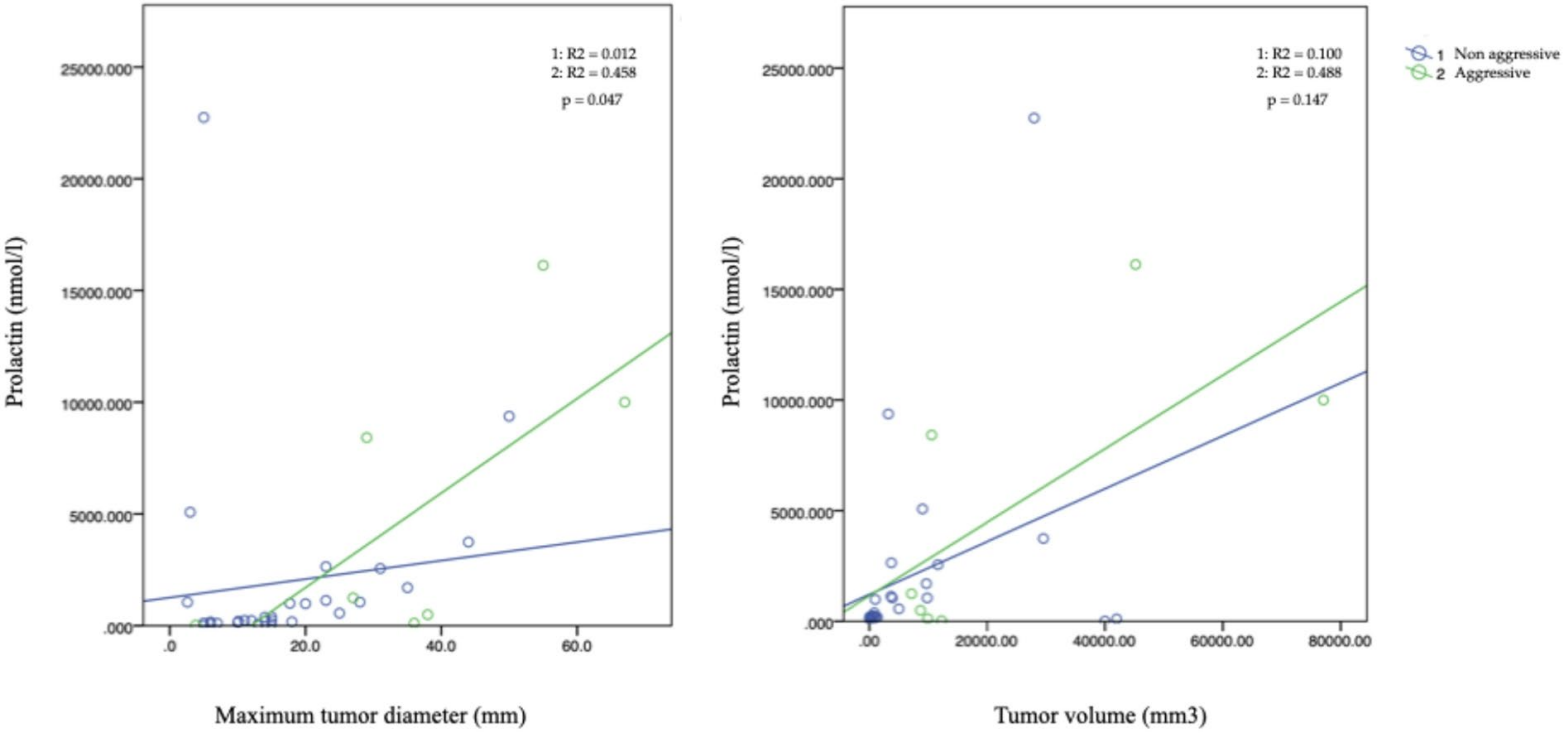
Correlation between baseline prolactin levels with maximum tumor diameter (left) and tumor volume (right) in aggressive and non-aggressive prolactinomas/lactotroph PitNETs.

Aggressive tumors had a larger maximum diameter (36 mm vs. 14 mm; *p* = 0.001), with higher rates of extrasellar extension (100% vs. 38.7%; *p* = 0.009), sphenoidal extension (90% vs. 38.7%; *p* = 0.007), cavernous sinus invasion (100% vs. 51.6%; *p* = 0.007), and bone invasion (40% vs. 3.2%; *p* < 0.001). All these associations remained statistically significant after age adjustment. A positive correlation was also observed between baseline serum prolactin levels and maximum tumor diameter, which was stronger in aggressive adenomas (*r* = 0.679; *p* = 0.047) (Fig. 2).

With regard to treatment, aggressive tumors showed greater resistance to medical therapy (70% vs. 22%; *p* = 0.003), required higher weekly cabergoline doses (2.9 mg vs. 2.1 mg; *p* = 0.005), and more frequently underwent surgery (80% vs. 16%; *p* = 0.005). These differences also remained significant after age adjustment. Transcranial approaches were exclusively performed in aggressive tumors (20% vs. 0%; *p* = 0.012). Surgical indications in aggressive tumors included medical resistance (30% vs. 6.4%; *p* = 0.003), visual disturbances (20% vs. 0%), and tumor apoplexy (10% vs. 0%). Reinterventions (30% vs. 0%; *p* = 0.002) and postoperative radiotherapy (40% vs. 6.5%; *p* = 0.004) were also significantly more frequent in aggressive tumors, persisting after adjustment for age.

### Long-term outcomes

One patient developed metastasis (metastatic PitNET/pituitary carcinoma) during follow-up. At the end of follow-up, 70% achieved normalized prolactin levels, with a median of 6.8 ng/mL (*p* = 0.040). Normalization of prolactin remained significant after adjustment for age. Additionally, 36% achieved tumor stability, 25% fulfilled criteria for aggressive progression, and 5% were in remission without treatment. No deaths were attributed to pituitary adenomas.

### Histopathological and immunohistochemical findings

**Fig. 3:**
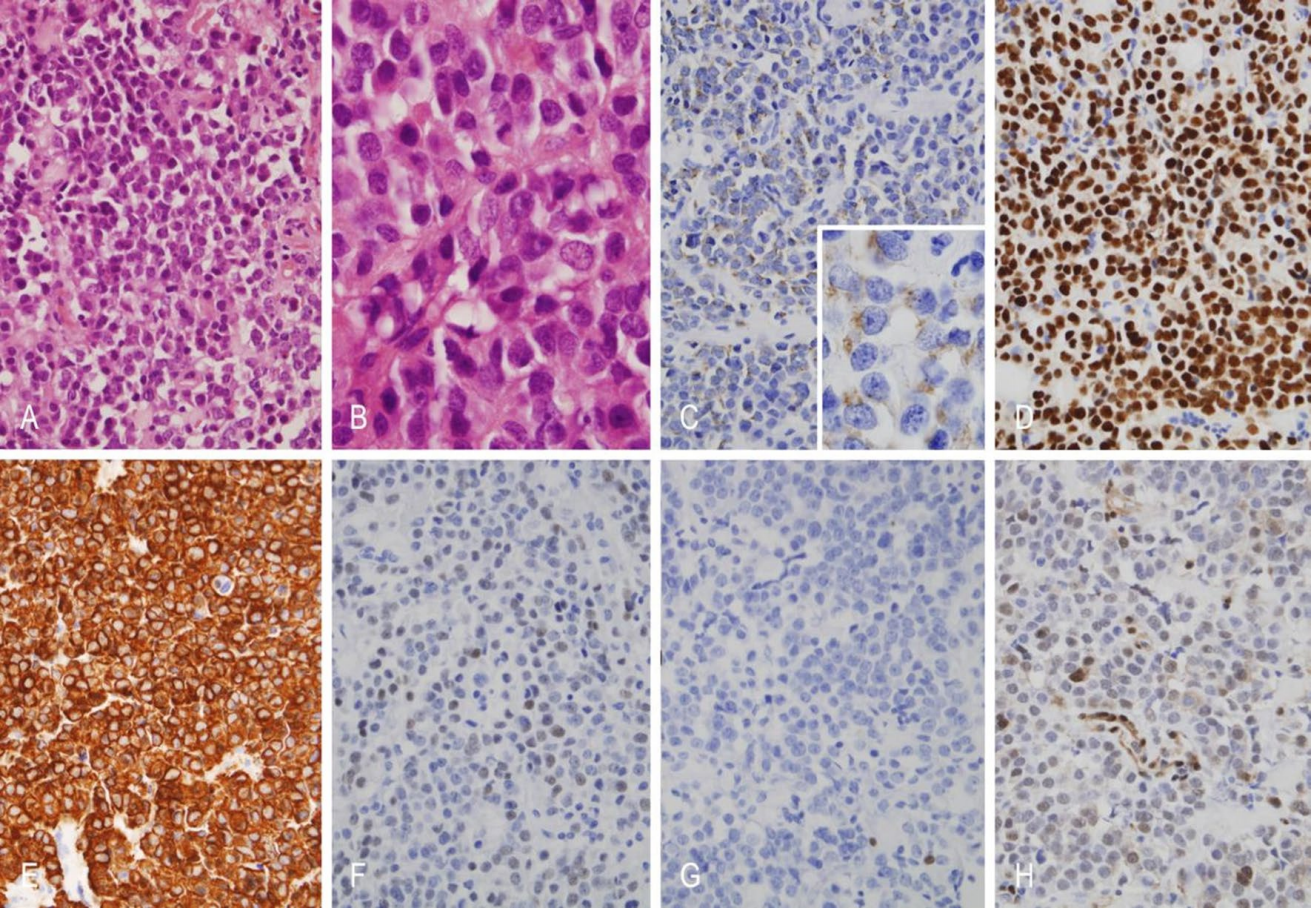
Microscopic features of case 1. (**A** and **B**) Lactotroph PitNET/adenoma with mostly chromophobic tumor cells, in this case arranged in sheets (hematoxilin and eosin stain). (**C**) Weak granular cytoplasmic immunoreactivity pattern for prolactin. (**D**) Diffuse nuclear immunoreactivity for PIT1. (**E**) Extensive cytoplasmic immunoreactivity pattern for keratins 8/18. (**F**) Nuclear reactivity for estrogen receptor alpha. (**G**) Ki67 proliferation index low (< 0.5%) (**H**) Conserved immunohistochemical expression for p27. Original magnification: A, 200X; B, 400X; C, 200X, inset, 400X; D-H, 200X.

**Fig. 4:**
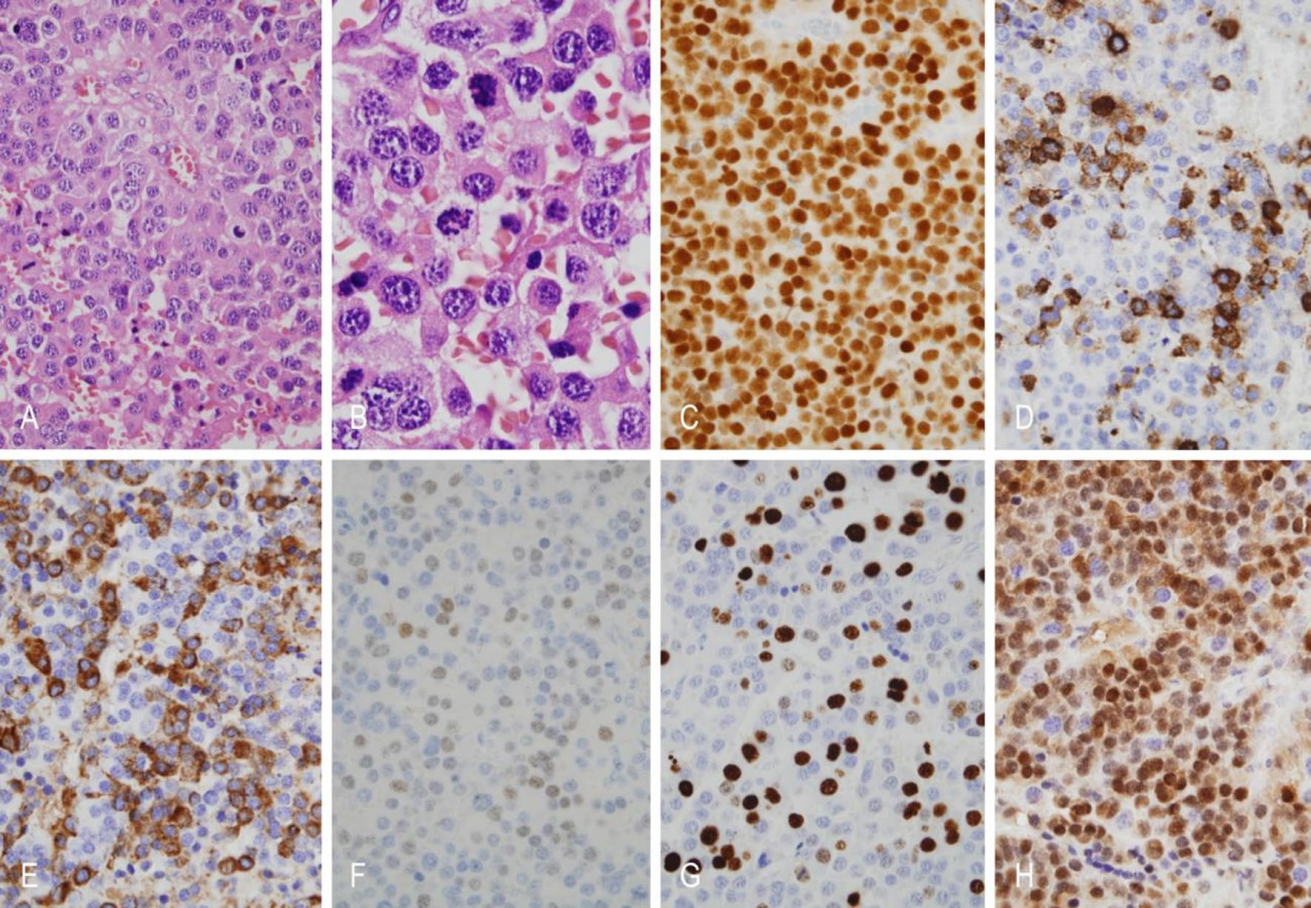
Microscopic features of case 4. (**A** and **B**) Metastatic PitNET with nuclear pleomorphism, mitoses (hematoxilin and eosin). (**C**) Diffuse nuclear immunoreactivity for PIT1. (**D**) Cytoplasmic immunoreactivity for GH. (**E**) Cytoplasmic immunoreactivity for PRL. (**F**) Nuclear reactivity for estrogen receptor alpha. (**G**) The Ki67 labeling index is high (22%) (**H**) Strong immunohistochemical expression of p27. Original magnification: A, 200X; B, 400X; C-H, 200X.

Histopathological and immunohistochemical analyses were available for four representative cases (Table 3). Regarding tumor subtype, three were classified as sparsely granulated adenomas and one as densely granulated. All tumors expressed p27 and displayed a wild type p53 staining pattern. Only one lost immunostaining for ATRX. PIT-1 expression was detected in three out of four cases. PRL in two and GATA3 in only one case. Keratin (CK8/18) immunoreactivity showed a peripheral cytoplasmic pattern in three tumors, while the metastatic PitNET was negative. The expression of estrogen receptors was decreased in all cases. The Ki-67 labeling index was high (22%) only in the metastatic PitNET. A somatic CDKN2A p. (Arg80) pathogenic variant (variant allele frequency: 96.6) was found in one case (see Figs. 3 and 4).

**Table 3:** Immunohistochemical and pathological features of representative cases of male patients with prolactinomas/lactotroph PitNETs.

Feature	Case 1 (PitNET)	Case 2 (aggressive PitNET)	Case 3 (PitNET)	Case 4 *(Metastatic PitNET)
Age	52	30	26	65
Tumor subtype	Sparsely granulated	Sparsely granulated	Densely granulated	Sparsely granulated
GATA3	–	+	–	–
Keratin (CK8/18)	Peripheral cytoplasmic reactivity	Peripheral cytoplasmic reactivity	Peripheral cytoplasmic reactivity	–
ERα	+	< 10%	< 10%	+
PIT1	+	–	+	+
GH	–	+	–	+
PRL	+	–	–	+
Mitosis	< 1/2 mm2	< 1/2mm2	< 1/2mm2	8/2mm2
Ki-67 labeling index	< 0.5%	< 0.5%	< 0.5%	22%
p27	+	+	+	+
p53	Wild-type pattern	Wild-type pattern	Wild-type pattern	Wild-type pattern
ATRX	-	+	+	+

* A somatic mutation of CDKN2A p.(Arg80) was identified in this tumor.

## Discussion

In our study of 41 prolactinomas/lactotroph PitNETs, the rate of aggressiveness was as high at 25%. In a case series of 36 males with prolactinomas/lactotroph PitNETs by Delgrande et al. invasiveness and aggressiveness were observed in 41% and 30% of cases, respectively^21^. It is important to note that the criteria used to classify aggressive adenomas have been modified in recent years^8^.

The mean age at diagnosis in our serie was 46.5 years, with variations reported in different studies ranging from 37 to 47 years^9,22–26^.

At the time of presentation, mean prolactin levels were 800 ng/ml, showing a wide variability compared to different studies, which have reported mean ranges of 99–14,393 ng/ml^23,24,26–28^. A positive correlation between prolactin levels and tumor size has been observed, which is also significantly demonstrated in this case series^23^. Prolactinomas/lactotroph PitNETs, in males are considered to be intrinsically more aggressive regardless of tumor size^26^.

Hypogonadism was highlighted as the most frequent clinical presentation (54%), followed by headache. These data area consistent with previous studies^22–24,29^. Patients with macroprolactinomas have a higher incidence of headache and visual abnormalities compared to patients with microadenomas^26,27^.

In addition to FSH/LH deficiency, TSH and ACTH deficiencies were among the most commonly observed findings, consistent with previous studies^25^. Other case series reported gonadal axis involvement in all male patients^3,26^. Overall, hypopituitarism was present in approximately three-quarters of the patients.

Aggressive prolactinomas/lactotroph PitNETs, compared to non-aggressive, presented with a higher frequency of visual disturbances, TSH and ACTH deficiency, which may be explained by higher rates of tumor volume, extrasellar and sphenoidal extension, invasion of the cavernous sinuses, and bone^24,30,31^. Additionally, the thyrotrophic and corticotrophic axes were more frequently affected in patients with macroprolactinomas, compared to microprolactinomas, as has been described previously^28,32^.

Most patients received primary medical treatment with DAs, while about a quarter of the patients required surgery, data consistent with the findings of other studies^9,25,26^. The most common indications for surgery, also in line with our series, were resistance and/or intolerance to DAs and/or extrasellar extension of the tumor^22,24^. In addition, patients who required surgical reintervention also received radiotherapy, a practice observed in other studies^9^.

Partial biochemical control was achieved at comparable rates in both non-aggressive and aggressive prolactinomas/lactotroph PitNETs. However, tumor control was more frequent in the aggressive group, likely reflecting the higher use of multimodal treatment strategies, including repeat surgery and postoperative radiotherapy. No mortality directly attributable to prolactinomas/lactotroph PitNETs was observed. One patient progressed to pituitary carcinoma, but death resulted from respiratory sepsis rather than tumor-related complications.

Pathological examination was limited by the small number of samples, and no differences were observed between prolactinomas/lactotroph PitNETs and aggressive cases. Although loss of p27 and p53 has often been associated with aggressive behavior^12,34,35^, all tumors in our series retained positivity for these markers, and ATRX loss was not detected in aggressive or metastatic PitNETs. The only metastatic case combined a markedly elevated Ki-67 index (22%) with a CDKN2A mutation, in line with previous studies that associate high Ki-67 values with aggressive biological behavior^21,33^. CDKN2A is a tumor suppressor gene in which high levels of mutations and LOH have been reported in prolactinomas and non-functional PitNETs^34^, and alterations in this gene have been linked to aggressive clinical behavior^34,35^; our findings seem to reinforce this relationship. Consistent with other studies, aggressive-invasive prolactinomas/lactotroph PitNETs were associated with unfavorable surgical outcomes, higher recurrence or progression rates during long-term follow-up, and elevated proliferative markers, including mitotic count > 10, Ki-67 > 5%, and frequent p53 positivity^36^.

This study has several inherent limitations related to its retrospective design. Reliance on medical records may have introduced biases in data collection, and the inability to control variables prospectively limits causal inference. Given the exploratory nature of the study and the limited number of eligible cases, no formal sample size calculation or power analysis was performed; instead, all available patients meeting inclusion criteria were included to maximize statistical power. Nevertheless, the relatively small sample size—particularly in the aggressive prolactinoma group—likely contributed to wide confidence intervals for variables such as visual disturbances and TSH/ACTH deficiencies, reflecting uncertainty in these estimates. Although age at diagnosis was adjusted for in multivariable analyses, residual confounding cannot be excluded, as other potentially important factors could not be systematically controlled due to incomplete historical data. In addition, the limited availability of pathological anatomy data further restricted the depth of analysis.

Despite these limitations, this study contributes valuable information to the relatively limited evidence on clinical predictors of aggressiveness in prolactinomas, with a particular focus on male patients. The direct comparison between aggressive and non-aggressive lactotroph PitNETs/prolactinomas within this subgroup, together with the relatively long follow-up period, provides meaningful insights into clinical outcomes and enriches the growing body of knowledge in this area.

## Conclusions

In our cohort, a quarter of prolactinomas/lactotroph PitNETs were classified as aggressive. Markedly elevated prolactin levels—closely correlated with tumor diameter—as well as visual disturbances and TSH/ACTH deficiencies should raise clinical suspicion of aggressive disease. Future studies should prioritize prospective designs and ideally involve multicenter collaborations to increase sample size, and ensure more standardized data collection. These approaches will be essential to validate our findings and to refine clinical predictors of aggressiveness in prolactinomas/lactotroph PitNETs.

## Data Availability

The datasets used and/or analyzed during the current study are available from the corresponding author upon reasonable request.
